# Effect of dietary supplementation of duckweed on growth performance, carcass and non-carcass traits of horro rams fed on a commercial-based diet

**DOI:** 10.1016/j.heliyon.2023.e17820

**Published:** 2023-06-29

**Authors:** Ashenafi Hunduma Gule, Debela Bayu Derese, Chala Merera Erge, Ulfina Galmessa Girgo, Hirpassa Kabeta Ejeta

**Affiliations:** a Department of Animal Science, School of Agriculture, Ambo University, P.O.Box 19, Oromia, Ethiopia; b Ethiopian Institute of Agricultural Research Center, P. O. Box 2003, Addis Ababa, Ethiopia

**Keywords:** Sheep, Commercial diet, Duckweed, Growth, Carcass

## Abstract

The study was aimed at evaluating growth performance, carcass, and non-carcass traits of Horro rams fed on commercial feed (CO) supplemented with different proportions of duckweed (DW). For the study, twenty-four yearling Horro rams initially weighing on average 17.73 ± 0.30 kg were used with a 15-day adaptation period (none-study period) and a 90-day study period. Each animal was randomly assigned to three treatments; T1 (100% CO), T2 (25% DW + 75% CO), and T3 (50% DW + 50% CO) with two replications each containing four animals. All diets were isocaloric and isonitrogenous and formulated according to the nutrient requirements of sheep. The data were analyzed using SAS. Results indicated that dry matter intake in T3 (914.37 g. d-1) was higher (P < 0.001) than in T1 (849.12 g. d-1) and T2 (870.50 g. d-1). Average daily body weight gain in T3 (77 g. d-1) was higher (P < 0.001) than in T1 (48.60 g. d-1) and T2 (58.50 g. d-1). Feed conversion efficiency was higher (P < 0.001) for T3 (0.08) followed by T2 (0.07) and T1 (0.05). Final body weight (24.56 kg), empty body weight (20.40 kg), hot carcass weight (10.27 kg), dressing percentage (42.01) and rib-eye muscle area (9.11 cm^2^) were higher (P < 0.001) for T3 than for others. Therefore, increasing the inclusion level of supplementary duckweed up to 50% improves growth performance, carcass quality, and yield of Horro ram.

## Graphical abstract

**Figure:**
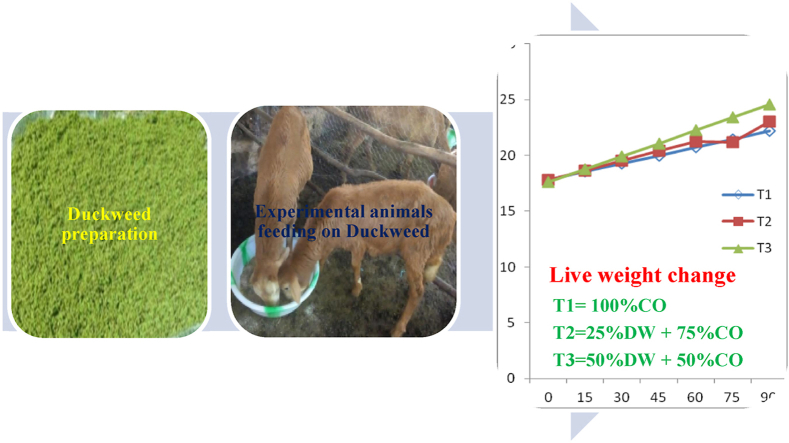

## 1 Introduction

Ethiopia has a potentially large sheep population in Africa; 40 million [1]; with above nine characterized breeds that play a great role in the socioeconomic development of the country [2]. These sheep breeds are characterized by different productive and reproductive capabilities [3]. For smallholder farmers in Ethiopia, sheep production is a suitable activity [4] for their greater environmental adaptability [5] and lower cost of investment [2]. In addition, sheep provide meat, skin, manure, and immediate cash income [6]. In Ethiopia, the demand-driven for animal products increases from time to time and, in contrast, the highest demand and accessibility for sheep meat is limited to annual festivals [7], attributed to the scarcity of protein feed supplements [8]. Horro sheep is one of the most widely distributed sheep breeds in the Western and Southwestern parts of Ethiopia by good production of meat (42% of carcass yield) and skin than other local sheep breeds. They are the most prolific sheep breed and have good weight: 2.80–2.90 kg at birth, 13–15 kg at weaning age, and 25–33.50 kg at one year. Irrespective of their large number [1] and genetic diversity [2], the productivity of sheep in Ethiopia is particularly credited to the poor quality and quantity of locally available feed resources and on top of that, the protein source feeds have become unaffordable for smallholder farmers due to its cost [9]. In this circumstance, it could be reasonable to look for other feeds which are affordable for the producers. Thus, duckweed is a potential protein alternative and a highly productive plant used as a feed [10].

Duckweed belongs to the family *Lemnaceae*, comprising five genera and thirty-six species [11], and it is probably the fastest-growing aquatic plant with almost exponential growth [12]. Though the yield and content of duckweed vary with species and cultivation medium [13], its yield ranges from 10 to 40 tons of dry matter per hectare per year. This compares with soybean producing less than 1 ton per year [14].

Duckweed grown under ideal conditions contains 17.50%–37.00% total protein on a dry weight basis [15], 59.33% of total carbohydrates, and 7.33% of lipids [16]. Duckweed has a wider range of essential amino acids [17] and is rich in macro and micronutrients [16]. Studies showed that duckweed is one of the potential protein sources that can be easily produced by farmers, and used as supplementary feed to livestock [18]. In Ethiopia, there is limited research or investigation about the partial or full replacement of costly commercial sheep feed using duckweed. Therefore, the current study is aimed at evaluating the growth performance, carcass, and non-carcass characteristics of Horro rams fed on commercial feed supplemented with duckweed.

## 2 Materials and methods

### 2.1 Description of the study site

The study was conducted at Ambo University, Guder Mamo Mezemir Campus, West Shoa Zone, Oromia Regional State, Ethiopia, located 124 km west of Addis Ababa. The study site is situated at 8°17′ N latitude and 37°1′ E longitude and at mid-altitude that ranges from 1380 to 3300 m above sea level. The mean annual rainfall of the area is 1079 mm, and the mean minimum and maximum daily temperatures of the area are 12 and 29 °C, respectively [19].

### 2.2 Production and preparation of duckweed

**Fig. 1:**
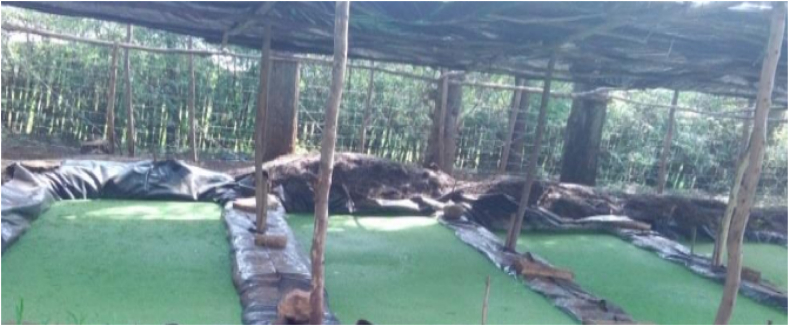
Duckweed production.

**Fig. 2:**
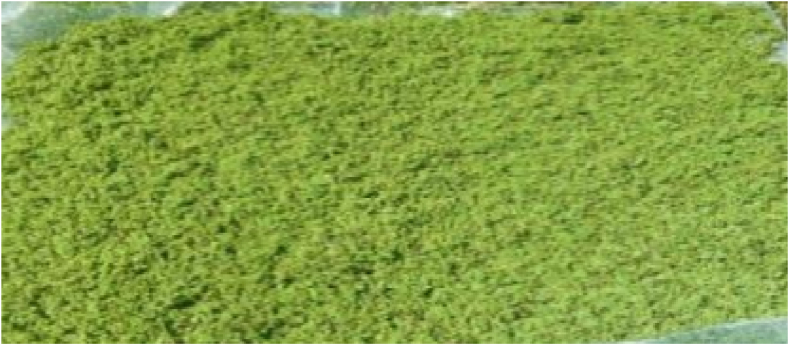
Spreading duckweed on wire sieve for partial drying.

Ten geo-membrane duckweed ponds with a plastic shade of 2 m × 5 m and 30 cm depth were prepared (Fig. 1), and 1.6 kg of duckweed per 1 m^2^ on a dry matter basis has been harvested. Harvested duckweed was spread out in sunlight for partial drying at 30% (Fig. 2) and then weighed by sensitive balance for feeding experimental sheep twice per day for 90 days of the experimental period.

### Ethical approval

The experiment was approved by Ambo University, Guder Mamo Mezemir Campus, Animal Scientific Research Ethical Committee No. ASRE/056/05/2022; dated May 03, 2022 as per the European Union directive number 2010/63/EU [20] for the care and use of animals for experimental and scientific purposes.

### 2.3 Experimental design and treatments

A total of twenty-four yearling Horro rams weighing 17.73 ± 30 kg were randomly assigned to three treatments (Table 1) of which each treatment had two replications (4 rams/replication). Generally, eight rams were randomly assigned to each dietary treatment in a completely randomized design. Commercial mixtures were formulated from 50% wheat bran, 30% nuog seed cake, 19% maize grain, and 1% salt. The diets were attempted to be formulated as isocaloric and isonitrogenous. In each treatment, experimental animals were fed twice a day; 400 g/head/day.

**Table 1:** Proportion of dietary treatments.

Experimental feed/head/day (%/g)	Treatment		
	T1	T2	T3
CO	100 (400)	75 (300)	50 (200)
DW	–	25 (100)	50 (200)

CO: Commercial feed; DW: Duckweed; T1: Treatment one; T2: Treatment two; T3: Treatment three.

### 2.4 Management of experimental sheep

Twenty-four-yearling Horro rams weighing 17.73 ± 0.30 kg were used. Body weight was taken after overnight fasting from the mean of two consecutive body weight measurements. They were vaccinated with the ovine pasteurellosis vaccine and anthrax vaccine, and also dewormed with albendazole bolus (300 mg) orally and ivermectin injection subcutaneously for internal parasites and sprayed with acarmic acaricide for external parasites early. After an adaptation period of 15 days (none-study period), the feeding trial lasted 90 days (study period).

### 2.5 Carcass evaluation

The carcass was evaluated after overnight fasting and slaughtering the animals. To slaughter, the animals’ jugular vein and carotid artery were severed using a knife after which blood was weighed. After slaughter, the skin was flayed and weighed. All internal organs, heads, and feet were removed and weighed. To estimate the hot carcass weight, the weight of legs below the hook and knee joints, thorax, head, abdominal, and pelvic cavity contents were removed.

To measure the rib-eye area of the carcass, the loin part was partitioned into fore and hind quarters between the 11th and the last ribs. The rib-eye area (in cm^2^) was measured at the 12th and 13th rib sites. Empty body weight has been determined by deducting the gut fill from the slaughter body weight, and the dressing percentage was calculated based on slaughter and empty body weight basis.

### 2.6 Chemical analysis of feed

Samples of duckweed and commercial mixture were analyzed for their DM (dry matter), CP (crude protein), and ash contents according to the methods of AOAC [21]. NDF (neutral detergent fiber) and ADF (acid detergent fiber) were analyzed according to the procedure of Van Soest and Robertson [22] at Holleta Agricultural Research Center, Ethiopia.

### 2.7 Dry matter intake

Dry matter intake (DMI) for each animal was calculated as.

DMI = CDMI + DDMI
Total dry matter intake (TDMI) was calculated as:
TDMI (% BW) = (DMI (g)/BW) × 100 and
TDMI (g**/**kg^0.75^) = DMI (g)**/**BW^0.75^

Where; CDMI = Commercial dry matter intake.

DDMI = Duckweed dry matter intake.

BW = Body weight.

#### 2.7.1 Body weight change (BWC)

The initial and final body weights of the rams were measured with the suspended weighing balance at the beginning and end of the experiment, respectively. Some of the measurements of body weight changes were calculated as:

BWC = Final body weight (FBW) – Initial body weight (IBW)

$$\text{Average}\;\text{daily}\;\text{gain}\;\left(\text{ADG}\right)=\frac{\text{FBW}-\text{IBW}}{\text{Number}\;\text{of}\;\text{feeding}\;\text{days}}$$

$$\text{Feed}\;\text{conversion}\;\text{efficiency}\;\left(\text{FCE}\right)=\frac{\text{ADG}\;\left(g\right)}{\text{Daily}\;\text{DMI}\;\left(g\right)}$$

### 2.8 Statistical analysis

To analyze the data, SAS version 9.0 [23], was used. To separate the treatment means, the least significant difference test was used. The model used is given below.

Yij = μ + τi + εij

Where Yij is the response variable.

μ is the overall mean,
τi is the treatment effect, and
εij is the random error

## 3 Results and discussion

The crude protein content of T3 (24.87%) was higher than T2 (20.66%) and T1 (16.45%) (Table 2). This was attributed to the high CP content of the supplementary duckweed (33.28%); which is higher than the crude protein content of duckweed grown under ideal conditions reported to contain 17.50–37.00% CP. Additionally, the energy content of the supplementary duckweed was 21.14%, which is lower than the 59.33% carbohydrate content of duckweed reported by Demann, [15]. But, another finding [24], reported a higher (38% CP) content of duckweed than the current finding, which confirms that the chemical composition of duckweed varies with the species of duckweed and cultivation medium [13]. The high CP (33.28%) content of the supplementary feed, duckweed indicates its high nutritive value and potential as a protein supplement to either the expensive conventional feed or poor quality feed.

**Table 2:** Chemical composition of the experimental diet and supplementary duckweed.

Chemical composition (% DM)	Experimental diet			Supplement feed
	T1 (Control)	T2	T3	DW
DM	90.03	86.10	82.17	74.32
OM	93.40	89.78	86.15	78.90
Ash	4.35	4.41	4.47	4.58
CP	16.45	20.66	24.87	33.28
NDF	42.03	45.87	49.72	57.41
ADF	23.43	29.22	35.02	46.62
CHO	41.56	36.46	31.35	21.14

DW: Duckweed; DM: Dry matter; OM: Organic matter; CP: Crude protein; NDF: Neutral Detergent fiber; ADF: Acid detergent fiber; CHO: Carbohydrate; T1 (control group): commercial 100% (400 g); T2: 75% commercial (300 g) + 25% duckweed (100 g); T3: 50% commercial (200 g) + 50% duckweed (200 g).

### 3.1 Feed intake

The daily dry matter intake (DMI) was greater (P < 0.001) in T3 (914.375 g/day) than in T2 (870.5 g/day) and T1 (849.175 g/day) (Table 3). This could be attributed to the treatment containing the increased level of duckweed supplement having reduced rumen retention time; thereby increasing the outflow rate and stimulating the intake than the other treatment groups. In addition, as the level of crude protein supplement increases, the dry matter intake increases; and so does the supply of nitrogen to the rumen microbes, having a positive effect on the rate of fermentation of the digesta [25]. On top of that, DMI can be positively or negatively affected by the physical and chemical characteristics of the feed [26]. The result of the current finding is higher than the DMI ranged 742.80–771.40 g/day reported by Gezahegn et al. [26], for the Bonga sheep fed on Noug seed cake substituted with mulberry and vernonia amygdalina. But, the result of the present study is slightly lower than the DMI ranged from 722.37 to 963.58 g/day reported by Mokonen et al. [27], for Salale sheep fed on natural grass supplemented with wheat bran and nuog seed cake. The variations may be attributed to variations in diet quality, environment, and body weight [28].

**Table 3:** Effect of dietary supplementation of duckweed on average dry matter and nutrient intake of Horro rams.

Intake (g/day)	Treatment				
	T1 (control)	T2	T3	SEM	SL
DMI	849.12^c^	870.50^b^	914.37^a^	3.688	***
DMI (g/kg^0.75^/day)	87.56	87.77	88.15	0.487	ns
DMI (% BW)	3.83	3.81	3.74	0.03	ns
CP	114.10^c^	128.60^b^	144.77^a^	0.274	***
OM	780.16	782.31	790.69	7.858	ns
ADF	326.95	343.84	355.98	23.15	ns
NDF	409.07^c^	434.70^b^	473.42^a^	2.1	***

T1 (control group): commercial 100% (400 g); T2: 75% commercial (300 g) + 25% duckweed (100 g); T3: 50% commercial (200 g) + 50% duckweed (200 g); DMI: Dry matter intake; BW: Body weight; OM: Organic matter; CP: Crude protein; NDF: Neutral detergent fiber; ADF: Acid detergent fiber; SL: Significance level; SEM: Standard error of the mean; a,b,c: Means with different superscripts in the same row differ significantly; **P < 0.01; ***P < 0.001; ns: non-significance.

The total dry matter intake as a proportion of percent body weight and per unit metabolic body weight basis have no variation (P > 0.05) across the three treatments. The current TDMI as a proportion of percent body weight ranged from 3.74 – 3.83%, which was slightly higher than the value of 2.48–2.55% BW for Begait lambs kept under different feeding options reported by Kahsu, [29]. The current result (3.74–3.83%) agrees with ARC [30] which recommends the range of dry matter intake for ruminants (3–5% of their body weight).

The dry matter intake per unit metabolic body weight (87.50–88.15 g/kg) obtained in the current finding agrees with the finding of Jalel [31] who recorded 76.20–85.90 g/kg of sheep.

The daily CP intake was higher (P < 0.001) for T3 (144.77 g/day) than for T1 (114.10 g/day) and T2 (128.60 g/day). Such variations in CP intake could be attributed to the differences in the CP content of the supplemented duckweed to the commercial feed. The current finding is in line with the finding of Zetina et al. [32] who reported supplementation of duckweed with Taiwan grass hay increases intake. According to ARC [30], the recommended level of CP required for a 20 kg growing lamb is 124 g/day.

The total NDF intake in T3 (473.42 g/day) was significantly higher (P < 0.001) than in T1 (409.07 g/day) and T2 (434.70 g/day), and the total OM and ADF intakes in T1, T2, and T3 have no significant variation (P > 0.05) for all treatments. The higher NDF in T3 may be due to its higher total dry matter intake. For both DMI and CP, the intake is higher in T3 and T2 than in the control group, and there is no refusal of experimental feeds. The study done by Darmy et al. [33] also found that when duckweed in dried and/or fresh form is fed to Meiron sheep, it could easily accept more effectively than when feeding urea. This could be attributed to the high crude protein content of duckweed leading to the high DMI.

### 3.2 Body weight change

The final body weight, body weight change, daily gain, and feed conversion efficiency were higher (P < 0.001) for T3 than for T2 and T1 (Table 4). This is probably because the supplementation of duckweed increases the supplementation of protein nutrients to improve the weight gain of rams. The current finding of average daily gain was slightly greater than the value of 42–50 g/d average daily gain of ram fed on non-conventional feed supplemented with commercial feed reported by Hagos et al., [34]. Studies showed a significant change in body weight gain when sheep feeding on low-quality feeds are supplemented with feeds high in either protein or energy [35,36]. In the current study, FCE was increased with the inclusion level of supplementary duckweed; which is high in crude protein content. This agreed with the idea that feed conversion efficiency increases with the ratio of diets that promote a high rate of body gain [29].

**Table 4:** Effect of duckweed supplementation on growth performances of Horro rams fed on a commercial-based diet.

Parameters	Treatment			SEM	SL
	T1 (Control)	T2	T3		
IBW (kg)	17.81	17.78	17.62	0.1126	Ns
FBW (kg)	22.51^c^	23.03 ^b^	24.56^a^	0.1989	***
BWC (kg)	4.70^c^	5.25^b^	6.94^a^	0.1561	***
ADG (g)	48.60^c^	58.30^b^	77.07^a^	1.7376	***
FCE	0.05^c^	0.07^b^	0.08^a^	0.0017	***

a–c: Means with different superscripts in the same row differ significantly; ** = (P < 0.01); *** = (P < 0.001); ns: non-significance; IBW: Initial body weight; FBW: Final body weight; BWC: Body weight change; ADG: Average daily gain; FCE: Feed conversion efficiency; SL: Significance level; SEM: Standard error of the mean; T1: 100% commercial mixture (400 g); T2: 75% commercial mixture (300 g) + 25% duckweed feed (100 g); T3: 50% commercial mixture (200 g) + 50% duckweed feed (200 g).

The feed conversion efficiency of the current study (0.05–0.08) was comparable with (0.05–0.04) and slightly higher than (0.03–0.09) reported by Gebrekidan [37], and Haymanot [38], respectively for Begait sheep fed on natural grass supplemented with concentrate feed. The variations could be attributed to the difference in breed, feed quality, and environmental and animal conditions.

### 3.3 DW: duckweed

**Fig. 3:**
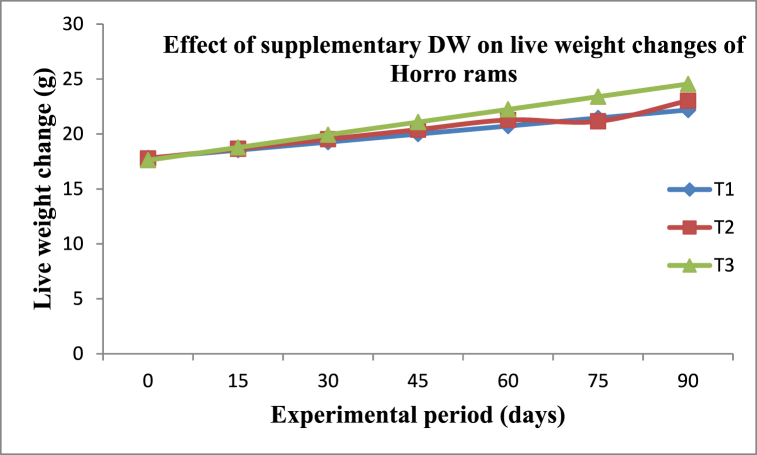
Effect of supplementary duckweed on live weight of Horro rams fed on commercial-based ration.

Sheep fed on an increased level of duckweed (50%) supplemented diet are greater (P < 0.001) in slaughter body weight (24.43 kg), empty body weight (20.40 kg), hot carcass weight (10.27 kg), dressing percentage (DP) and rib-eye muscle area (9.11 cm^2^) than T1 and T2 (Fig. 3 and Table 5). Sheep in the control group had the lowest slaughter weight compared with the duckweed-supplemented groups (Fig. 3). The increase in slaughter weight with the supplementation of high-protein feed was also reported by Atti et al., [39]. The highest level of the duckweed-supplemented group (T3) resulted in a higher hot carcass weight, which is probably due to relatively more muscle development than others. Dressing percentage values on the slaughter weight basis in the supplemented group are significantly higher (P < 0.01), which agrees with the values of Allam et al. [40], and Abdel [41].

**Table 5:** Effect of supplementary duckweed on carcass characteristics of Horro rams fed on a commercial-based diet.

Parameters	Treatments				
	T1 (Control)	T2	T3	SEM	SL
Slaughter body weight (kg)	20.34^c^	22.28^b^	24.43^a^	0.262	***
Empty body weight (kg)	16.45^c^	17.90^b^	20.40^a^	0.275	***
Hot carcass weight (kg)	7.92^b^	8.65^b^	10.27^a^	0.198	***
Rib-eye muscle area (cm^2^)	6.08^c^	7.11^b^	9.11^a^	0.1776	***
Dressing percentage on:					
Slaughter weight basis	38.84^b^	38.90^b^	42.01^a^	0.662	**
Empty body weight basis	48.14	48.38	50.31	0.8635	ns

a–c: Means with different superscripts in the same row differ significantly; * = (P < 0.05); ** = (P < 0.01); *** = (P < 0.001); ns: Non-significance; SL: Significance level; SEM: Standard error of the mean; T1: 100% commercial mixture (400 g); T2: 75% commercial mixture (300 g) + 25% duckweed feed (100 g); T3: 50% commercial mixture (200 g) + 50% duckweed feed (200 g).

Dressing percentages on an empty body weight basis are greater than on a slaughter weight basis, showing the influence of gut fill on dressing percentage. Thus, judging carcass weight based on live weight at slaughter may not be suitable. The dressing percentage on an empty body weight basis is not different (P > 0.05) between the supplemented and control groups. The dressing percentage based on empty body weight is 48.14%, 48.38%, and 50.31% in T1, T2, and T3, respectively. The current finding is in line with the finding of Ewnetu [42], for Menz and Horro sheep.

The rib-eye muscle area is 6.08 cm^2^, 7.11 cm,^2^ and 9.11 cm^2^ for T1, T2, and T3, respectively. As to Mathios et al. [43], and Wondesn [44], supplementation of poor-quality feed with high-quality feed had a positive effect on the rib-eye muscle area. The greater (P < 0.001) rib-eye muscle areas were obtained in the higher supplementation of duckweed. The relatively higher rib-eye muscle area in duckweed-supplemented animals might be due to high weight gain, which is related to the higher crude protein content of the duckweed.

### 3.4 Edible offal

In the current finding, duckweed supplementation did not distress the weight of blood, heart, and tongue (P > 0.05) (Table 6). The other edible components were improved (P < 0.001) upon higher supplementation with duckweed (T3). The TEO (total edible offal) of supplemented rams with duckweed (T3) is greater (P < 0.001) than other treatments. This indicates that the improvement in TEO due to supplementation for sheep-fed commercial mix with duckweed (T3) is closely associated with body weight gain and differences in their energy or dietary protein intake level. The TEO stated as a percentage of slaughter weight in this experiment is 19.26%, 18.52%, and 17.64% for T1, T2, and T3, respectively. Takele [45] also recorded the improved edible offals of Horro lambs fed on duckweed-supplemented ration.

**Table 6:** Effect of supplementary duckweed on slaughter weight-based proportion of edible offal of Horro rams fed on a commercial-based diet.

Parameters (%)	Treatment				
	T1(Control)	T2	T3	SEM	SL
Heart	0.60	0.60	0.59	1.9594	ns
Liver	1.66^b^	1.70^b^	1.91^a^	3.2792	***
Kidney	0.29^c^	0.33^b^	0.36^a^	0.4118	***
Kidney fat	0.17^b^	0.18^b^	0.29^a^	0.9242	***
Omental fat	0.53^c^	0.55^b^	0.60^a^	0.9453	***
Tongue	0.90	0.90	0.80	0.5266	ns
Empty gut	7.91^b^	7.92^b^	8.05^a^	9.03	***
Tail	1.56^c^	2.42^b^	2.81^a^	8.42	***
TEO	19.22^c^	20.26^b^	21.19^a^	25.69	***

a–c: Means with different superscripts in the same row differ significantly ** = (P < 0.01); ***= (P < 0.001); ns: Non-significance; SL: Significance level; SEM: Standard error of the mean; T1: 100% commercial mixture (400 g), T2: 75% commercial mixture (300 g) + 25% duckweed feed (100 g) and T3: 50% commercial mixture (200 g) + 50% duckweed feed (200 g).

### 3.5 Inedible offal

The weight of total inedible offals (TIO) are not influenced (P > 0.05) with duckweed supplementation (Table 7). In the current study, supplementing duckweed increased (P < 0.001) spleen and pancreas, and esophagus weight. In the current study, no difference (P < 0.001) in gut fill was recorded for all treatments; which could be attributed to the time taken (16-h fasting period) which causes equal shrinkage of their gut fill.

**Table 7:** Effect of supplementary duckweed on slaughter weight-based proportion of inedible offal of Horro rams fed on a commercial-based diet.

Inedible offal (%)	Treatment				
	T1 (Control)	T2	T3	SEM	SL
Blood	5.66	0.76	5.68	19.239	ns
Gut fill	21.82	21.77	22.44	151.4	ns
Spleen and pancreas	0.24^c^	0.28^b^	0.32^a^	0.7086	***
Testis and penis	1.15	1.13	1.12	1.051	ns
Esophagus	2.02^c^	2.24^b^	2.57^a^	7.470	***
Skin and leg	10.98	11.55	12.32	50.02	ns
Head	9.22^b^	9.40^a^	9.51^a^	9.01	*
TIO	45.43	46.36	48.28	201.1	ns

a–c: Means with different superscripts in the same row differ significantly; * = (P < 0.05); ** = (P < 0.01); *** = (P < 0.001); ns: Non-significance; SL: Significance level; SEM: Standard error of the mean; TIO: Total Inedible offal; T1: commercial feed 100% (400 g); T2: 75% commercial feed (300 g) + 25% duckweed feed (100 g); T3: 50% commercial feed (200 g) + 50% duckweed feed (200 g).

## 4 Conclusions

The study conducted to evaluate growth performance, carcass and non-carcass traits of Horro ram fed on commercial feed supplemented with duckweed showed that, with duckweed-supplemented commercial feed up to 50% level, it is possible to increase growth performance, quality, and yield of carcass traits of Horro ram, and thus could increase rate of return. Supplementary duckweed increases protein content of the ration with low cost to achieve improved feed intake, fast growth rate and body weight gain, and thus facilitate fattening practice of Horro ram. This indicates the possibility of using duckweed as an alternative protein feed resource to bridge the gap of feed scarcity and quality, especially in Ethiopia. Thus, for the further recommendation of this feed, testing for the presence of anti-nutritional factors has also an additional importance.

## Author contribution statement

Ashenafi Hunduma: Conceived and designed the experiment; Performed the experiment; Analyzed and interpretated the data and Wrote the paper.

Debela Bayu: Conceived and designed the experiment; Analyzed and interpretated the data and Wrote the paper.

Chala Merera: Conceived and designed the experiment; Contributed reagents, materials, analysis tools or data and Wrote the paper.

Ulfina Galmessa: Conceived and designed the experiment, Analyzed and interpretated the data and Wrote the paper.

Hirpessa Kabeta: Analyzed and interpretated the data and Wrote the paper.

## Data availability statement

Data included in article/supp. Material/referenced in article.

## Declaration of interest's statement

The author declares no conflict of interest.

## Additional information

No additional information is available for this paper.

## Declaration of Competing Interest

The authors declare that they have no known competing financial interests or personal relationships that could have appeared to influence the work reported in this paper.
